# Statement of Retraction

**DOI:** 10.1080/22221751.2021.1917762

**Published:** 2021-04-22

We, the Editors and Publishers of *Emerging Microbes and Infections*, have retracted the following article:

Shi-Lei Dong, Wei-Lin Hu, Yu-Mei Ge, David M Ojcius, Xu'ai Lin & Jie Yan (2017) A leptospiral AAA+ chaperone–Ntn peptidase complex, HslUV, contributes to the intracellular survival of *Leptospira interrogans* in hosts and the transmission of leptospirosis, *Emerging Microbes & Infections*, 6:1, 1-16, DOI: 10.1038/emi.2017.93

This article is being retracted due to reuse of images from other articles by the same authors, representing different results.

This includes a histopathology panel from Figure 7A (Wild-type 3d/Kidney), which can be found in the following article, where it is presented as Figure 5 (ΔMPI/Kidney 7d), with slightly altered rotation and staining:

Yu-Mei Ge, Ai-Hua Sun, David M Ojcius, Shi-Jun Li, Wei-Lin Hu, Xu’ai Lin, Jie Yan, M16-Type Metallopeptidases Are Involved in Virulence for Invasiveness and Diffusion of Leptospira interrogans and Transmission of Leptospirosis, The Journal of Infectious Diseases, Volume 222, Issue 6, 15 September 2020, Pages 1008–1020, DOI: 10.1093/infdis/jiaa176

In addition, a Leptospira cell staining panel from Figure 7E (7 d/ΔhsIUB) was found to have been presented as a result from an earlier experiment, within urine of a different strain of hamsters, within Figure 5D (7(100x)/ Wild-type strain) of the following article:

Kokouvi Kassegne, Weilin Hu, David M. Ojcius, Dexter Sun, Yumei Ge, Jinfang Zhao, X. Frank Yang, Lanjuan Li, Jie Yan, Identification of Collagenase as a Critical Virulence Factor for Invasiveness and Transmission of Pathogenic Leptospira Species, The Journal of Infectious Diseases, Volume 209, Issue 7, 1 April 2014, Pages 1105–1115, DOI: 10.1093/infdis/jit659

When approached for an explanation, the authors were unable to provide an explanation or verify their original data. The authors wish to apologize for their errors and the resulting inconvenience. The authors agree with the decision to retract this article.

We have been informed in our decision-making by our policy on publishing ethics and integrity and the COPE guidelines on retractions.

The retracted article will remain online to maintain the scholarly record, but it will be digitally watermarked on each page as “Retracted”.

